# Management and disposal of polythene bag waste among urban households in Lira City: a cross sectional survey

**DOI:** 10.1186/s12889-026-27146-1

**Published:** 2026-03-30

**Authors:** Sam Orech, Cecilia Adyero, Charles Okolimong, Evaline Ajwang, Ruth Anne Akello

**Affiliations:** 1 Department of Community and Public Health, Busitema University, Mbale City, Uganda; 2 Department of Environmental Health Sciences, Lira University, Lira City, Uganda; 3 Faculty of Education, Team University, Kampala, Uganda

**Keywords:** “Knowledge”, “management”, “disposal”, “polythene bag waste”, “Lira City”

## Abstract

**Background:**

Improper disposal of waste has implications for public health worldwide. This issue is not limited to use polythene bags alone. Notably, over 53% of solid wastes are generated in urban areas, especially within households. Managing solid wastes including polythene bags, used as daily shopping bags, requires strict interventional measures. There is porosity in executing these measures. This study, therefore, assessed knowledge, attitude, and practices of disposing used polythene bags among residents of Lira City.

**Methods:**

A cross-sectional survey was employed. Quantitative data using structured questionnaires were collected among 421 randomly selected residents of Lira City. Data was analysed using STATA Version 17, summarized as frequencies, percentages, means, and standard deviations. Pearson Chi-square test was run for bivariate analysis, and multivariate analysis using logistic regression at 95% CI, and a p-value of 0.05 to obtain significant variables associated with the outcome variable.

**Results:**

Overall, the response rate was 99.8%. Even then, more than half of the respondents had poor practices of disposal (52.5%). Yet a good number of the respondents 42.5% were aged 18 to 28 years, female 64.9%, and 34.9% attained a primary level of education. Although most of the respondents were self-employed 41.1%, married 61.3%, and Catholic 35.2%, the majority 78.9% had good knowledge of proper disposal of used polythene bags. Significantly, age [AOR = 3.007; 95% CI; 1.074–8.417], Sex [AOR = 1.2; 95% CI; 0.82–1.97], Reduced Injuries [AOR = 1.2; 95% CI; 0.38–4.15], Drainage blockage [AOR = 3.04; 95% CI; 2.00-4.63], and Human Health Problems [AOR = 1.7; 95% CI; 1.15–2.73] were associated with disposal of used polythene bags among residences in Lira City.

**Conclusion:**

Despite high levels of knowledge, improper disposal of used polythene bags remained common practices among urban households in Lira City, significantly influenced by age and perceived risks related to drainage blockage and human health. Targeted community education focusing on younger residents and strengthened enforcement of existing waste management bylaws are recommended.

**Supplementary Information:**

The online version contains supplementary material available at 10.1186/s12889-026-27146-1.

## Background

Globally, solid waste generation is increasing, with polythene bags leading the way. In Sub-Saharan Africa, plastic waste generation is influenced by a variety of factors, including urbanization [1]. As of 2019, the population of Sub-Saharan Africa was estimated to be approximately 1 billion [2], and the amount of waste generated was 180 million tonnes at the rate of 0.5% per capita per day, with 70% openly dumped and 17 million tonnes of the plastic waste generated annually [3].

In Africa more than 15 countries are taking the plastic bag problem very seriously by either imposing a ban or charging huge taxes on them [4]. These countries include Kenya, Mali, Cameroon, Tanzania, Uganda and Ethiopia. The efficacy of the bans and taxes is hard to determine and concretize because there is still a black market for plastic bags, and people continue to use them illegally [5]. Used polythene bags waste from urban cites contributes to the world’s 1.3 billion tons of waste generated annually, which is expected to increase to 2.2 billion tons by 2025 [6].

In Uganda, like in many other developing countries, typically one to two-thirds of the solid waste generated is not collected [7]. More than 53% of Uganda’s solid waste is generated in urban centers from households [8]. As a result, uncollected solid waste, including the polythene bags, end up in rivers, tunnels, and other water ways, causing blockage [9]. Poor disposal of polythene bags contributes to flooding and the breeding of insects and rodent vectors, with over two million birds and animals dying every year due to eating polythene bags or as a result of chocking [10].

The government of Uganda has developed a number of legal and institutional frameworks to address the challenges of solid waste management across urban towns and cities. The constitution of the Republic of Uganda (1995), Article 245 (a), provides measures intended to protect and preserve the environment from abuse, pollution and degradation [11], thereby establishing the constitutional foundation for environmental management and public health protection. In addition, there are the National Environment (Waste Management) Regulations, S.I. No 52/1999; the Local Government Act 1997, and solid waste management ordinances [12]. All these legal frameworks have provisions on how waste should be properly managed to protect and conserve the environment [13].

Additionally, Uganda has attempted to ban plastic bags usage, but implementation has been inconsistent because of lobbying by manufacturers, disagreement among politicians, and a lack of public awareness about the need to rigorously implement and adhere to the ban. In April 2015, despite numerous demonstrations from members of private sector, including court battles, the Ministry of Water and Environment, under National Environment Management Authority (NEMA), put into action the ban on importation, manufacture, and use of polythene bags of gauge below 30 microns [14]. The implementation, however, has not been smooth, as it affected businesses and other government agencies, including the cabinet, which fought the ban and call for its suspension, leaving the public in a state of indecision. Others continue to use the banned substance despite threats of legal action from NEMA [15]. Countries like Australia as a whole don’t have a ban on plastic bags; however, several states and territories have begun to put bans in place, including the Northern Territory, South Australia, and Tasmania and this approach can be adopted by Lira city authorities [16].

In addition to solving the problem in Uganda, Kampala Capital City Authority (KCCA) has made efforts to provide street dustbins and conduct garbage collection more frequently in places where plastic litter can prevent the flow of waste water. According to Jean Marie Takouleu 2021, Coca cola has also signed a memorandum of understanding with Care and Assistance for Forced Migrants (CAFOMI) to encourage recycling and increase what people earn from collecting plastic waste from $0.05 per kilogram to $0.13 [17]. According to the Nile Post 2019, in another attempt to reduce the problem of the polythene bag litter, an entrepreneur called Aweko Faith has come up with an innovative way to transform discarded plastic bags into backpacks for everyday use through her organization, Transform Africa, based in Mpigi, Uganda [18].

Lira city is one of the newly created cities in the country, with an increasing population growth rate estimated to reach 596,900 people from the current 478,500 by 2030. The population density is highest around the urban and peri-urban areas within Lira, according to the District National Development Plan (NDP) III 2021 [19].

Literature shows that indiscriminate disposal of used polythene bags is associated with poor knowledge, attitude, and practices, especially at household level. A study in Kenya showed that more than 50 per cent of household dwellers with sheep and goats lacked knowledge about the danger of plastic bags to their animals [20]. Relatedly, lack of cost-effective disposal alternative methods and weak enforcement of existing laws on waste disposal, combined with limited knowledge and negative attitude constrained coastal cities in Sir Lanka from effectively disposing of plastic polythene waste [21].

As a result of indiscriminate dumping of solid waste, including polythene bags, especially in urban towns and cities [22], drainage channels are blocked, resulting in flooding, while water logging caused by used polythene bags act as breeding cites for insects and rodent vectors. This increases the prevalence of diseases such as malaria and diarrhoeal diseases, in addition to risking the lives of animal, with 60% dying as a result of consumption [23]. According to the City Environment Officer, annually approximately 22,000 tonnes of solid waste are generated annually in Lira, with 23.4% being plastic waste. About 17,000 tonnes are disposed of, while the rest remain uncollected and currently there no documented literature on the disposal of used polythene waste in the city [24]. This study, therefore, assessed the knowledge, attitude, and practices of disposing used polythene bags among residents of Lira City, hence contributing to the achievement of Uganda Vision 2040 and the Sustainable Development Goals (SDG 11) [25].

## Materials and methods

### Study design

A cross-sectional analytical survey was employed, in which quantitative data were collected. A cross-sectional survey was conducted to assess polythene bag disposal practices among urban households. This design enabled efficient collection of quantitative data to determine prevalence and identify associated factors.

### Study area

The study was carried out in Lira city, which has 2 divisions; Lira City East and Lira City West. The city has 7 Wards, where Lira City East has Railways, Adekokwok, Ngetta, and Central, while Lira City West division has Adyel, Ojwina and Lira wards. The study was conducted in four wards within the two divisions; Adyel, Lira, Central, and Adekokwok.

Lira City is located in the Lango sub-region in northern Uganda. Physically the district lies between latitudes 1^0^ 21’ and 2^0^ 42’ north of the Equator and longitudes 2^0^ 53’ and 3^0^ 37’ east of Greenwich. The city is bordered by the districts of Lira in the north, Agago also in the north, Otuke in the northeast, Alebtong in the east, Dokolo in the south, Kwania in the southwest, Kole in the west, Oyam and Gulu in the northwest. The district covers approximately a total area of 1,326 km2, of which 1286.22 km2 is land area. The other 39.78 square kilometers constitute forest reserves, wetlands and geomorphic landforms. The city is 368kms from Kampala City in the central part of Uganda. Subsistence agriculture, animal husbandry and commercial fishing from area lakes constitute the economic engine of the district.

Therefore, it’s worthy to conduct a study in this setting, in order to support environmental sustainability and urban health, thus aligning with the national goals under Uganda’s Vision 2040 and SDG 11.

### Source population

The source population comprised all residents of Lira City.

### Study population

The study population consisted of residents of Lira City who met the study inclusion criteria and were selected to participate in the study during the data collection period.

### Eligibility criteria

#### Inclusion criteria

All residents above 18 years of age, with a sound mind staying in households (HHs) for at least 6 months within the selected ward, participated in the study.

#### Exclusion criteria

All residents above 18 years of age who decline to consent, had an ill health condition, or health worker were excluded from the study.

### Determining the sample size

The sample size was determined using the Kish Leslie (1965) formula for the calculation of sample size [26]. Calculated from the formula;

$$n=\frac{{z}^{2}PQ}{{d}^{2}}$$

Where;

n= Estimated sample size

z = the standard deviation corresponding to a 95% confidence interval, usually 1.96

p = the prevalence of people with knowledge, attitude and practices on disposal of used polythene bags, (Since there is limited information on a related study done, 50% was assumed for maximum variation)

q = 1-p, d = 0.05, the acceptable margin of error at 95% confidence interval. *n* = 1.96*1.96*0.5*0.5/0.05*0.05

= 3.841*0.25/0.0025

= 0.96025/0.0025

= 384 participants

Therefore, when a 10% non-response rate is considered and the sample size was 422 participants for this study.

### Sampling procedure

Probability sampling methods were used, focusing on two clusters at the city divisions; East and West. A list of all 7 wards was generated, and a simple random sampling by ballot technique with replacement was used to select 50% of the wards, enhancing representativeness, reducing intra-cluster correlation [27]. Two (2) wards were picked from each division. From the 4 wards, lists of all the parishes in each ward were then generated, and using simple random sampling, two (2) parishes in each ward were selected. At the parish level, lists of all the villages/cells were also generated, and simple random sampling by ballot technique was then used to select two (2) villages/cells from each parish. At the village/cell level, sampling proportionate to size and systematic sampling was employed using a table of random numbers of households using an interval of five (5) from the Village register to obtain respondents to take part in the study (Table 1).

**Table 1:** Sampling procedure to obtain the respondents

Divisions	Wards	Parishes	Villages /Cells	Respondents
East and West	Simple random sampling by Ballot technique	Simple random Sampling	Simple random sampling by Ballot technique	Systematic Sampling using Random number Table
4 wards	Adyel	2 Parishes	2 villages/cells	106
	Railways	2 Parishes	2 villages/cells	105
	Central	2 Parishes	2 villages/cells	106
	Lira	2 Parishes	2 villages/cells	105
Total	**4 selected Wards**	**8 selected Parishes**	**8 selected villages/cells**	**422 respondents**

Selecting 50% of the wards ensured adequate representation of socio-demographic and environmental variability across the two city divisions while reducing cluster-level sampling error. In multistage cluster sampling, especially where the number of primary sampling units was limited, sampling a larger proportion (30–50%) of clusters improves the reliability and generalizability of estimates [28, 29]. This approach also allowed equitable representation of both divisions while remaining feasible within available resources.

### Data collection procedures

Primary data were collected using quantitative methods, where structured questionnaires were digitized in KoBoCollect, and face-to-face interviews were conducted. Research assistants comprising of health assistants and community development officers, were comprehensively trained on data collection tools, procedures, and quality control measures. Before data collection, the tools was pre-tested in a small population to identify and fixed potential issues in data collection. The principal investigator conducted data validation on daily basis until data collections was complete.

### Data collection tools

The structured questionnaires used in this study were developed by the author based on the objectives of the study. The questionnaires had four main sections for data collection, information on: (1) household and demographic characteristics; (2) Knowledge on disposal of used polythene waste; (3) Attitude of the respondents on used polythene waste disposal, and (4) Used Polythene waste disposal practices [30].

### Study variables

#### Dependent variables

The practices were measured using nine items assessing household disposal behaviors. Each correct practice was scored 1 and each incorrect practice 0, yielding a total score of 0–9. The score was dichotomized using Bloom’s 70% cut-off: respondents scoring ≥ 7 were classified as having proper disposal practices, while those scoring < 7 were classified as having improper disposal practices. The outcome variable was coded as 1 = proper practices and 2 = improper practices.

#### Independent variables

Independent variables included (socio-demographic and household characteristics), (Knowledge), and (attitude) of the respondents toward polythene bag waste disposal.

Socio-demographic and household characteristics included age, sex, marital status, education level, occupation, and household size.

Knowledge of polythene bag waste disposal was assessed using structured knowledge questions. Correct responses were scored 1 and incorrect or “don’t know” responses 0. Composite knowledge scores were categorized as good knowledge (≥ 70%) or poor knowledge (< 70%).

Attitude toward polythene bag waste disposal was measured using 5-point Likert-scale statements ranging from strongly disagree (1) to strongly agree (5). Negatively worded items were reverse-coded. Total attitude scores were summed and classified as positive attitude (≥ 70%) or negative attitude (< 70%) using Bloom’s cut-off.

### Data quality management

To ensure data quality, the structured questionnaires were pretested prior to data collection, and necessary revisions were made to improve clarity and consistency. Data collectors received training on the study objectives, ethical considerations, and proper administration of the questionnaires. KoBoCollect was configured with built-in validation checks, including skip patterns and range restrictions, to minimize entry errors during data collection. Daily reviews of submitted data were conducted to check for completeness, consistency, and accuracy, and any identified discrepancies were addressed promptly through follow-up with the data collectors.

### Data management and analysis

#### Data management

Data were collected using interviewer-administered structured questionnaires programmed in KoBoCollect and uploaded electronically to a secure server. The collected data were then downloaded and exported to Microsoft Excel for initial review and coding. Subsequently, the dataset was exported to STATA version 17.0 for data cleaning, management, and statistical analysis.

#### Data analysis

Data analysis was done using STATA version 17.0. Univariate analysis was used to generate proportions and percentages using measures of central tendency; that is mean, median and mode. Bivariate analysis was used to find out associations between the dependent and independent variables, where chi square test was used for categorical variables. Multivariate analysis was used to determine the several independent variables associated with outcome variable using logistic regression at 95% confidence interval, *p* ≤ 0.05.

### Ethical considerations

Ethical approval was thought from the Gulu University Research Ethics Committee (GUREC) (GUREC-2021-119). An introduction letter was also obtained from Lira University, Faculty of Public Health that helped to start the study processes. Permission to conduct the study was obtained from the office of the City Clerk and Environment officer before proceeding to the Divisions and Wards to commence the data collection. All procedures were conducted in accordance with the ethical principles of the 1975 Declaration of Helsinki. A written informed consent was obtained from all the participants following a detailed explanation of the study, including potential risks and participant rights. Confidentiality and anonymity were strictly upheld; all data were de-identified, and access was restricted to the research team.

## Results

### Socio demographic characteristics of respondents

Table 2 shows that majority of the respondents were aged 18 to 28 years, 179 (42.5%); female, 273(64.9%); and had primary level of education 147(34.9%). Most of the respondents were self-employed, 173(41.1%); married, 285(61.3%); and catholic, 165(35.2%) (Table 2).

**Table 2:** Socio demographic characteristics the respondents (*N* = 421)

Variables	Frequency (*n*)	Percentages (%)
Age		
18–28 years	179	42.5
29–39 years	106	25.2
40–50 years	70	16.6
51–61 years	41	9.7
62–75 years	25	5.9
Sex		
Male	148	35.2
Female	273	64.9
Household Head		
Yes	252	59.9
No	169	40.1
Level of Education		
None	62	14.7
Primary	147	34.9
Secondary	116	27.6
Post-secondary/Tertiary level	96	22.8
Occupation		
Peasant Farmer	89	21.1
Business person/self employed	173	41.1
Professional	63	15.0
Housewife	31	7.4
Not employed	65	15.4
Religion		
Catholic	165	39.2
Protestant	140	33.3
Pentecostal	80	19.0
Muslim	23	5.5
Others	13	3.1
Marital Status		
Single	85	20.2
Divorced	44	10.5
Married	285	61.3
Widowed	24	5.7
Cohabiting	10	2.4

#### Practices of disposal of used polythene bags among residents of Lira city

**Fig. 1:**
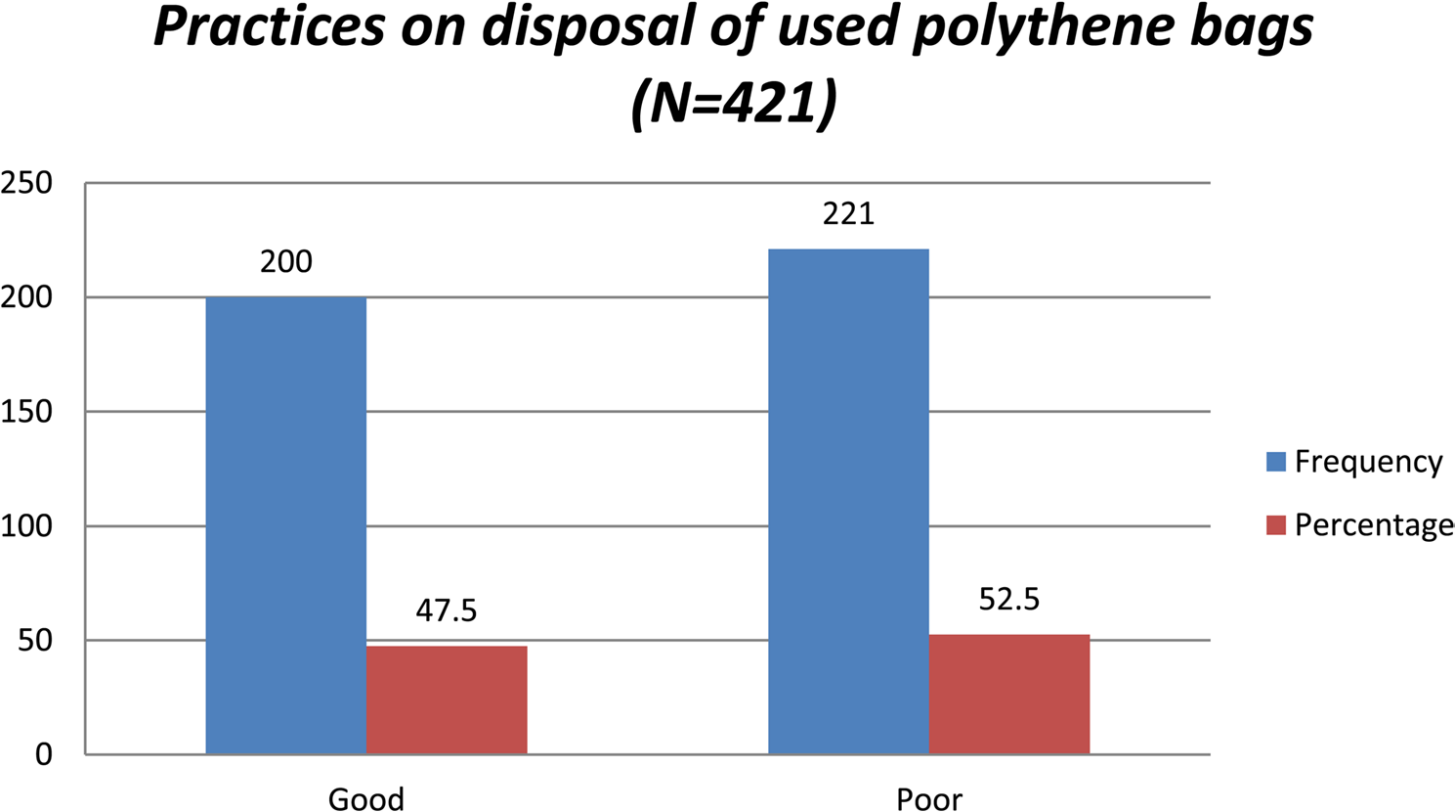
Practices on disposal of used polythene bags

Figure 1 show that more than half, 221(52.5%) of the respondents had poor practices of disposal of used polythene bags (Fig. 1).

#### Knowledge of respondents of towards disposal of used polythene bags

**Fig. 2:**
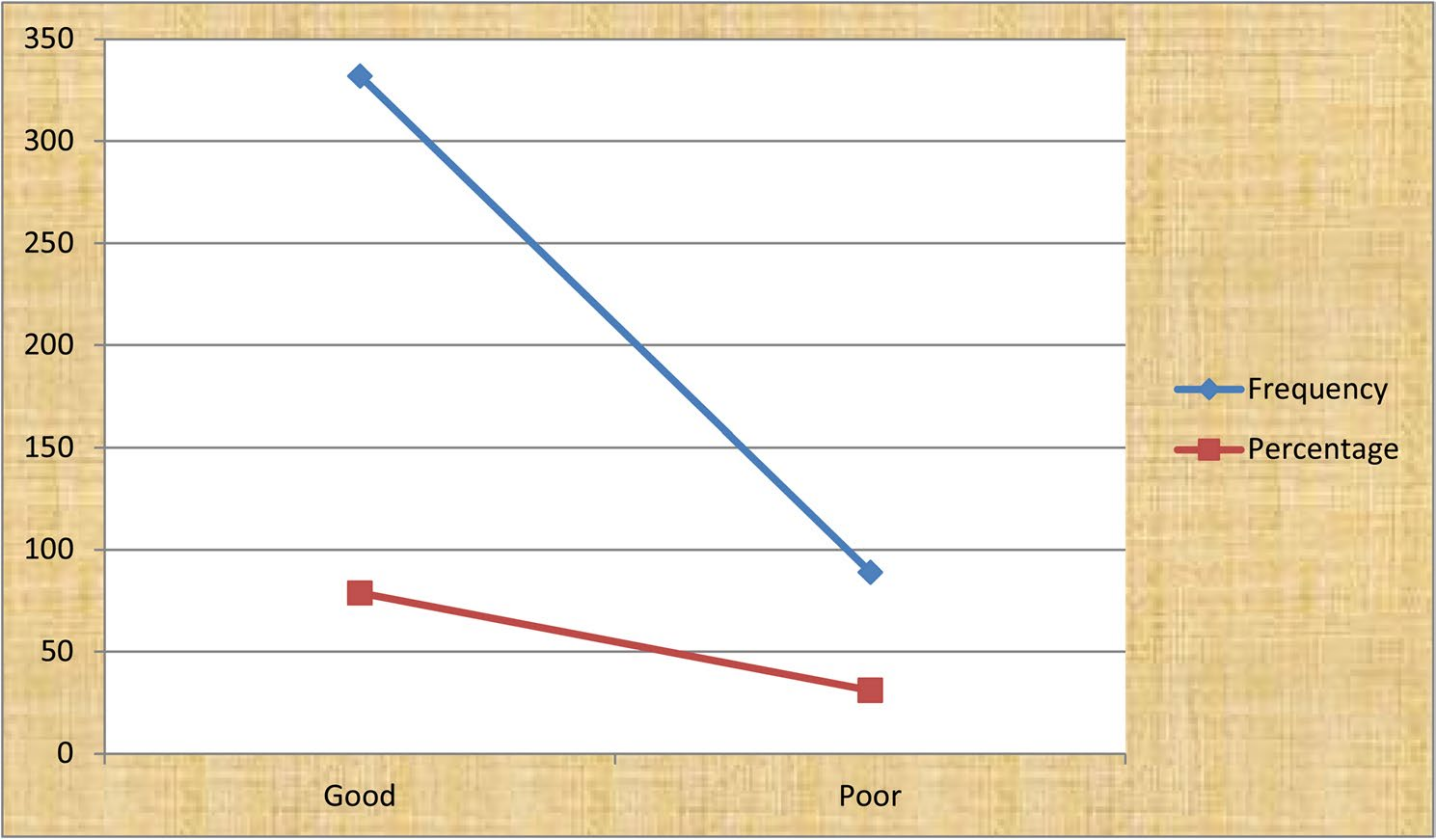
Knowledge of respondents of towards disposal of used polythene bags

Figure 2 show that the majority of the respondents (78.9%) had good knowledge about disposal of used polythene bags (Fig. 2).

#### Attitudes of respondents on disposal of polythene bags

Majority of the respondents did not segregate waste, 360(85.5%). Most of the respondents reported that polythene bags lead to drainage blockage, 42.8% (180/421); destruction of the environment, 96.4% (406/421); and human health problems, 54.9% (231/421). Most of the respondents reported that waste segregating is not important (Table 3).

**Table 3:** Attitudes of respondents regarding polythene bag disposal (*N* = 421)

Variable	Frequency (*n*)	Percentage (%)
Segregate waste		
Yes	61	14.5
No	360	85.5
Important to segregate waste		
Easy identification, disposal and burning		
Yes	36	8.6
No	385	91.5
Food residue given to animals as feeds		
Yes	2	0.5
No	419	99.5
Avoid environmental pollution		
Yes	8	1.9
No	413	98.1
Reduce injuries and accidents		
Yes	15	3.6
No	406	96.4
Dangers of poor disposal of polythene bags		
Animal death		
Yes	93	22.1
No	328	77.9
Drainage blockage		
Yes	180	42.8
No	241	57.2
Destroys environment		
Yes	406	96.4
No	15	3.6
Human health problems		
Yes	231	54.9
No	190	45.1

### Association between socio demographic variables, knowledge and attitudes with disposal practices of used polythene bags

At the bivariate analysis, sex and level of education showed a positive association with appropriate disposal practices of used polythene bags at a significance threshold of *p* < 0.20. Knowledge was not significantly associated with disposal practices at this level of analysis. However, several attitudinal factors were significantly associated with disposal practices, including perceptions related to avoiding environmental pollution (*p* = 0.05), prevention of drainage blockage (*p* < 0.001), reduction of human health problems (*p* = 0.02), reduction of injuries and accidents (*p* = 0.13), and the belief that food residues in polythene bags could be given to animals as feed (*p* = 0.18), as in Table 4.

**Table 4:** Results of a chi square test between socio demographic variables, knowledge attitudes and their practice towards disposal of used polythene bags *n* = 421

Variable	Practice		Chi square	*P* value
Age	**Good**	**Poor**		
18–28 years	88(49.2)	91(50.8)	3.504	0.48
29–39 years	53(50)	53(50)		
40–50 years	34(48.6)	36(51.4)		
51–61 years	17(41.5)	24(58.5)		
62–75 years	8(32)	17(68)		
Sex				
Male	78(52.7)	70(47.3)	2.472	0.12*
Female	122(44.7)	151(55.3)		
Household Head				
Yes	122(48.4)	130(51.6)	0.207	0.65
No	78(46.2)	91(53.8)		
Level of Education				
None	25(40.3)	37(59.7)	4.218	**0.24**
Primary	64(43.5)	83(56.5)		
Secondary	61(52.6)	55(47.4)		
Tertiary level	50(47.5)	46(47.9)		
Occupation				0.87
Peasant Farmer	38(42.7)	51(57.3)	1.228	
self employed	85(49.1)	88(50.9)		
Professional	31(49.2)	32(50.8)		
Housewife	14(45.2)	17(54.8)		
Not employed	32(49.2)	33(50.8)		
Religion				
Catholic	73(44.2)	92(55.8)	1.572	0.81
Protestant	67(47.9)	73(52.1)		
Pentecostal	41(51.3)	39(48.8)		
Muslim	12(52.2)	11(47.8)		
Others	7(53.8)	6(46.2)		
Marital Status				
Single	37(43.5)	48(56.5)	5.254	0.26
Divorced	27(61.4)	17(38.6)		
Married	121(46.9)	137(53.1)		
Widowed	12(50)	12(50)		
Cohabiting	3(30)	7(70)		
Knowledge				
Good	155(46.7)	177(53.3)	0.423	0.52
Poor	45(50.6)	44(49.4)		
Segregate waste				
Yes	31(50.8)	30(49.2)	0.314	0.58
No	169(46.9)	191(53.1)		
Important to segregate waste				
Easy identification, disposal and burning				
Yes	20(55.6)	16(44.4)	1.023	0.31
No	180(46.8)	205(53.2)		
Food residue given to animals as feeds				
Yes	0	2(100)	1.818	0.18*
No	200(47.5)	219(52.3)		
Avoid environmental pollution				
Yes	1(12.5)	7(87.5)	4.01	0.05*
No	199(48.2)	214(51.8)		
Reduce injuries and accidents				
Yes	10(66.7)	5(33.3)	2.89	0.13*
No	190(46.8)	216(53.2)		
Dangers of poor disposal of polythene bags				
Animal death				
Yes	41(44.1)	52(55.9)	0.559	0.45
No	159(48.5)	169(51.5)		
Drainage blockage				
Yes	112(62.2)	68(37.8)	27.307	< 0.001*
No	88(36.5)	153(63.5)		
Destroys environment				
Yes	195(48.0)	211(52.0)	1.253	0.26
No	5(33.3)	10(66.7)		
Human health problems				
Yes	122(52.8)	109(47.2)	5.783	0.02*
No	78(41.1)	112(58.9)		

* Significant variable at *p* < 0.20

### Factors associated with used polythene bag disposal practices among residents

Variables with a *p*-value ≤ 0.20 at the bivariate analysis were entered into a multivariable logistic regression model to adjust for potential confounding. A backward stepwise elimination approach was applied, whereby variables with *p*-values > 0.05 were sequentially removed until a final model comprising only statistically significant factors was obtained. The goodness of fit of the final model was assessed using the Hosmer–Lemeshow test, which indicated an adequate model fit (*p* = 0.07).

Increasing age was independently associated with appropriate disposal practices of used polythene bags. Older respondents demonstrated more than threefold higher odds of proper disposal compared to younger respondents. Specifically, participants aged 62–75 years were significantly more likely to practice proper disposal (AOR = 3.01; 95% CI: 1.07–8.42; *p* = 0.036).

In addition, attitudinal factors were significantly associated with proper disposal practices. Awareness of drainage blockage was strongly associated with appropriate disposal (AOR = 3.00; 95% CI: 2.00–4.63; *p* < 0.001), as was concern about human health problems (AOR = 1.77; 95% CI: 1.16–2.73; *p* = 0.009) (Table 5).

**Table 5:** Factors associated with practices of disposal of used polythene bags among residents of Lira City *N* = 421

Practice	COR	95% CI	*P* value	AOR	95% CI	*p*-value
Age						
18–28	1.00			1.00		
29–39	0.967	0.598–1.563	0.89	0.846	0.514–1.392	0.511
40–50	1.024	0.589–1.779	0.93	1.232	0.675–2.247	0.496
51–61	1.365	0.687–2.715	0.37	1.836	0.881–3.828	0.105
62–75	2.05	0.844–5.004	0.13	3.007	1.074–8.417	0.036*
Sex						
Male	1.00			1.00		
Female	1.379	0.923–2.060	0.12	1.278	0.827–1.976	0.269
Reduce injuries						
Yes	1.00			1.00		
No	2.273	0.764–6.769	0.14	1.271	0.389–4.156	0.692
Drainage blockage						
Yes	1.00			1.00		
No	2.864	1.921–4.269	< 0.001	3.044	2.00-4.633	< 0.001*
Human health problems						
Yes	1.607	1.090–2.368	0.02	1.00		
No				1.777	1.156–2.734	0.009*

* Significant variable at *p* < 0.05

## Discussion

### Socio demographics characteristics of the respondents

The study results show that majority of the respondents were aged 18 to 28 years, 179 (42.5%); female, 273(64.9%); and had primary level of education, 147(34.9%). Most of the respondents were self-employed, 173(41.1%); married, 285(61.3%); and catholic, 165(35.2%). The findings are in line with those from another study, which shows that women buy basic consumer goods such as food, health items, clothing and household products more often than men. Men more often buy expensive goods like cars and electronic equipment. The results of this current study is also in line with a study conducted in Nairobi and Kajiado Counties, Kenya which indicate that irrespective of peoples’ gender, age, education, or occupation, majority of the population frequently used plastic bags in their daily operations but does not dispose them properly [20].

### Practices of the residents on disposal of used polythene bags

Findings from this study indicate generally poor practices regarding the disposal of used polythene bags. More than half of the respondents (52.5%) demonstrated improper disposal practices, which is largely explained by structural and service-related gaps. Notably, 85.8% (361/421) of respondents reported the absence of designated waste collection points within their areas of residence, and an equal proportion indicated that no private waste collection companies operated in their communities. Among the few respondents (*n* = 60) who reported the presence of waste collection services, the majority (37/60) cited door-to-door collection, suggesting limited and inconsistent coverage.

Regarding waste storage practices, the majority of households stored waste on the ground (59.4%), while only small proportions used bags (16.4%), dustbins (15.4%), or improvised containers such as baskets or cut jerrycans (6.7%). These practices reflect inadequate access to appropriate waste storage materials and organized waste management systems.

These findings are consistent with previous studies reporting poor waste disposal practices despite adequate knowledge. For example, Shahzahi et al. reported that over 50% of respondents had poor waste disposal practices, attributing this to limited awareness campaigns and the unavailability of public dustbins [20]. Similarly, a study in India found that 27% of respondents practiced open dumping due to lack of resources, even though they possessed adequate knowledge of proper waste disposal [31]. In the Philippines, low levels of solid waste management practices were reported among students despite relatively high levels of knowledge and positive attitudes [32]. Together, these studies support the notion that knowledge alone is insufficient to translate into appropriate disposal practices without enabling infrastructure and services.

### Knowledge of the residents on disposal of used polythene bags

In this study, majority 78.9% of respondents demonstrated good knowledge regarding the proper disposal of used polythene bags.

This finding is comparable to studies conducted elsewhere, including South Africa, where approximately 80% of community members were knowledgeable about household waste disposal and aware of the environmental and health consequences of improper disposal [33]. Similar findings have been reported in Kenya, where about half of the respondents were knowledgeable about the negative effects of improper disposal of polythene bags on animals and the environment [34]. Evidence from Nigeria also indicates that knowledge of solid waste management is significantly associated with educational attainment [35]. The relatively high knowledge levels observed in the present study may be attributed to household-level sensitization campaigns and community outreach initiatives, as reported by respondents during interviews, where residents are encouraged to use locally available materials such as buckets and sacks for temporary waste storage.

However, despite high levels of knowledge, proper disposal practices remained poor, and knowledge was not significantly associated with disposal practices at the multivariate level. This finding suggests that knowledge, although necessary, is not a sufficient predictor of proper disposal behavior in the absence of supportive infrastructure, enforcement mechanisms, and accessible waste management services.

### Attitude of the residents on disposal of used polythene bags

With respect to attitude, the majority of respondents (85.5%) reported that they do not segregate waste at the household level. Nevertheless, respondents demonstrated awareness of the consequences of improper disposal, with many acknowledging that it leads to environmental destruction (96.4%), drainage blockage (42.8%), and human health problems (54.9%). These findings are consistent with studies conducted in Pakistan, where over half of respondents reported that improper household waste disposal contributes to environmental contamination and increased disease burden [20].

Despite these relatively negative perceptions toward improper disposal, attitude was also not significantly associated with proper disposal practices in the multivariate analysis. This indicates that favorable attitudes do not necessarily translate into improved practices, particularly in settings where waste segregation facilities, collection points, and routine collection services are lacking.

### Factors associated with used polythene bag disposal

At the multivariate level, age and sex of respondents were significantly associated with proper disposal of used polythene bags. Older respondents had more than 3-fold increased odds of proper disposal compared to younger respondents. This finding aligns with a study conducted in Kazakhstan, which identified age as an independent predictor of participation in solid waste separation, with individuals aged over 45 years demonstrating more positive waste management behaviors [36]. In contrast, studies from Nairobi and Kajiado Counties in Kenya reported poor disposal practices across demographic groups, irrespective of age, gender, or education [37, 38]. The observed association between older age and proper disposal in the present study may be explained by cumulative exposure to sensitization efforts, greater environmental awareness, and experience with past public health campaigns.

Importantly, although knowledge and attitude were included as independent variables in the analytical model, neither showed a statistically significant association with proper disposal practices after adjustment for confounders. This finding underscores the dominant role of structural, service-related, and contextual factors such as availability of collection points and waste collection services over individual cognitive factors in shaping disposal practices.

### Strengths and limitations of the study

This study employed a large and representative urban household sample with a high response rate, enhancing internal validity. The use of multistage probability sampling, electronic data collection, and multivariable logistic regression strengthened data quality and control for confounding. The knowledge–attitude–practice framework enabled a comprehensive assessment of polythene bag waste management with clear public health and policy relevance.

However, the cross-sectional design precludes causal inference. Disposal practices were self-reported and may be subject to social desirability and recall bias. The dichotomization of practice scores may have masked behavioral variation, and findings are limited to an urban setting, restricting generalizability to rural populations.

## Conclusion and recommendations

Despite high levels of knowledge, improper disposal of used polythene bags remained common among urban households in Lira City, with practices significantly influenced by age and perceived risks related to drainage blockage and human health. The absence of functional waste collection points and limited coverage of private waste collection services emerged as major barriers to appropriate disposal practices.

Targeted community education focusing on younger residents, expansion of accessible waste collection points, establishment and equitable distribution of designated waste collection points, and strengthened enforcement of existing waste management bylaws are recommended. Public–private partnerships and incentives for recycling and reuse should be prioritized to improve household disposal practices and urban environmental health in line with SDG 11 and Uganda Vision 2040.

Future longitudinal and mixed-methods studies are needed to assess causal relationships and evaluate the effectiveness of behavior change and policy interventions on household polythene bag disposal practices.

## Supplementary Information

Supplementary Material 1.

## Data Availability

Data is available on request from the corresponding author on email: [samorech62@gmail.com] (mailto:samorech62@gmail.com).

## Abbreviations

UBOS: Uganda Bureau of Statistics
DHS: Demographic Household Surveys
DHO: District Health Officer
USMID: Uganda Support to Municipal Infrastructure Development Program
PPW: Plastic Polythene Waste
GOU: Government of Uganda
SWM: Solid Waste Management
NEMA: National Environment Management Authority
NDP: National Development Plan
KCCA: Kampala Capital City Authority
UN-OCHA: United Nations Office for Coordination of Humanitarian Affairs
SPSS: Statistical Package for Social Sciences
GHG: Green House Gases
