# Supplementary material for: Management and disposal of polythene bag waste among urban households in Lira City: a cross sectional survey
Source: BMC Public Health. 2026 Mar 30;26:1510. doi: 10.1186/s12889-026-27146-1 (PMC13154551; doi:10.1186/s12889-026-27146-1)
Supplement: Supplementary file 1 — Supplementary Material 1. [file 12889_2026_27146_MOESM1_ESM.pdf]

Appendix 2: Questionnaire

SECTION 1: PRELIMINARY SECTION

Name of Respondent

Surname…………………………..First Name:………………………………………

Date and time of Interview:…………………………………………………………..

Area of Residence (Tick):

Ojwina		Ngetta		Lira		Adyel	
Railways		Central		Adekokwok			

Ward:…………………………………………………………………………………….

Do you agree to give an informed consent to participate in this research as a respondent and agree that data you will provide can be used for the purpose of this research?

Yes:……………… No:……………….

**Socio-demographic information**		
	**Question**	**Response**
A01	What is your date of birth? (Estimate using provided age and birthday)	
A02	Sex of respondent	1. Male<br>2. Female
A03	Position in the Household.	
A04	What is your level of education? (Select one)	1. Primary<br>2. Secondary<br>3. Post-secondary/Tertiary level<br>4. None

A05	What is your occupation?	1. Peasant farmer<br>2. Professional eg Banker<br>3. Business person<br>4. Others (specify)
A06	What is your religion?	1. Catholic<br>2. Pentecostal<br>3. Protestant<br>4. Muslim<br>5. Others (specify)
A07	What is your marital status?	1. Single<br>2. Divorced<br>3. Married<br>4. Widowed<br>5. Cohabiting
A08	How many people live in this household?	1. Total Males<br>2. Total Females
**Knowledge on used polythene bags disposal among households.**		
B01	Do you generate waste in your household?	1. Yes<br>2. No
B02	What types of waste do you generate in your household? Select all applicable	1. Food Residues<br>2. Plastics<br>3. Polythene bags<br>4. Metal<br>5. Broken Glass<br>6. Liquid Waste
B03	What is the estimated quantity of solid waste you generate per day?	1. 5-10kgs<br>2. 10 – 20kgs<br>3. 30-50kgs<br>4. 50kgs and above
B04	How do you dispose of the generated waste?	1. Burning<br>2. Open space dumping<br>3. Composting<br>4. Collect in sacks awaiting trucks to pick for disposal
B05	Do you use polythene bags in your household?	1. Yes<br>2. No

B06	How often do you buy things packed in polythene bags?	1. Daily<br>2. Weekly<br>3. Once a month<br>4. Twice a month
B07	How many pieces of polythene bags do you use in a week?	1. 1-4<br>2. 5-10<br>3. 10-20<br>4. 20-30<br>5. ≥30
B08	What do you use the polythene bags for?	1. Carrying items from the market<br>2. Lighting charcoal<br>3. Covering food while cooking<br>4. None of the above
B09	How do you dispose used polythene bags in your household?	1. Burning<br>2. Indiscriminate disposal<br>3. Incinerate<br>4. Mix with other solid waste
**Attitude of households on disposal of used polythene bags**		
C01	Do you segregate waste in your household?	1. Yes<br>2. No
C02	If yes, how do you segregate	1. Plastics alone<br>2. Metal alone<br>3. Polythene alone<br>4. Food residues alone<br>5. Glass alone
C03	Why do you think it's important to segregate waste?	
C04	Why do you use polythene bags in your household?	1. Low price<br>2. Given for free from the market<br>3. Light weight<br>4. Lack alternative materials<br>5. Durability
C05	Why do you dispose of the polythene the way you do in your household?	
C06	Do you know of any dangers of improper disposal of used polythene bags?	1. Animal death<br>2. Blockage of drainage channels

		3. Destruction of natural beauty of environment<br>4. Human health problems
C07	Do you think there would be any other alternative packing materials to polythene?	1. Yes<br>2. No
C08	If yes, which one?	
**Practices on disposal of used polythene bags**		
D01	Do you have any designated waste disposal facility/point by the city authority in your area?	1. Yes<br>2. No
D02	If yes, Is it easily accessible for your household?	1. Yes<br>2. No
D03	If yes, how often is the generated waste collected from the final collection Centre before final disposal?	1. Daily<br>2. Once a week<br>3. Twice a week<br>4. Monthly<br>5. Never
D04	Do you pay for the waste disposal services	1. Yes<br>2. No
D05	If yes, how much do they charge?	1. 1,000/= – 5,000/=<br>2. 6,000/= - 10,000/=<br>3. 11,000/= – 15,000/=<br>4. Above 15,000/=
D06	How often is the payment done?	1. Daily<br>2. Weekly<br>3. Bi weekly<br>4. Monthly
D07	How do you store the segregated waste in your household?	1. Dust bin<br>2. Plastic Bags<br>3. On the ground<br>4. Buckets/Jerricans
D08	Who is responsible for disposal of waste in your household?	1. Adults<br>2. Children<br>3. Both adults and children
D09	Are there private companies that collect waste from	1. Yes

	this area?	2. No
D10	How do they operate?	1. Door to door services<br>2. Have one collection point<br>3. Random
D11	How often do they come to collect the waste?	1. Daily<br>2. Weekly<br>3. Monthly<br>4. Once in two months
End of the questionnaires, thanks for your time		
